# Facile Synthesis of Mixed-Mode Weak Anion-Exchange Microspheres via One-Step Pickering Emulsion Polymerization for Efficient Simultaneous Extraction of Strongly and Weakly Acidic Drugs from Reservoir Water

**DOI:** 10.3390/polym12092089

**Published:** 2020-09-14

**Authors:** Xiaoyi Gou, Yun Li, Chaonan Huang, Xiuhua Zhu, Jiping Chen

**Affiliations:** 1 School of Materials Science and Engineering, Dalian Jiaotong University, Dalian 116028, China; gxy@dicp.ac.cn; 2 Key Laboratory of Separation Sciences for Analytical Chemistry, Dalian Institute of Chemical Physics, Chinese Academy of Sciences, Dalian 116023, China; liyun@dicp.ac.cn; 3 School of Environmental & Municipal Engineering, Qingdao University of Technology, Qingdao 266033, China; huangchaonan@qut.edu.cn; 4 School of Environmental and Chemical Engineering, Dalian Jiaotong University, Dalian 116028, China

**Keywords:** weak anion-exchange, mixed-mode SPE, Pickering emulsion polymerization, strongly and weakly acidic NSAIDs, reservoir water

## Abstract

Poly(2-(diethylamino)ethyl methacrylate-co-divinylbenzene) (poly(DEAEMA-co-DVB)) microspheres with mixed-mode weak anion-exchange (WAX) character were successfully fabricated for the first time via facile one-step Pickering emulsion polymerization. The obtained poly(DEAEMA-co-DVB) particles had good spherical geometry, uniform particle size in the range of 30–40 µm, a large specific surface area of 575 m^2^/g, and a pore size range of 5–30 nm, according to the SEM and nitrogen adsorption–desorption results. Using these mixed-mode WAX microspheres as packing material, a reliable and robust analytical method based on solid phase extraction and high performance liquid chromatography with ultraviolet detection (SPE-HPLC-UV) was developed for simultaneous determination of six strongly and weakly acidic nonsteroidal anti-inflammatory drugs (NSAIDs, niflumic acid, diflunisal, naproxen, ketoprofen, mefenamic acid, and diclofenac) in reservoir water. Under optimized conditions, it was applicable to preconcentrate up to 500 mL of reservoir water samples on the WAX cartridges with satisfying recoveries (88–96%) for all the NSAIDs tested. The limits of detection were in the range of 0.002–0.025 μg L^−1^, respectively. Our results showed that the developed mixed-mode WAX poly(DEAEMA-co-DVB) phase containing a tertiary amine with a pKa value of approximately 10.7 could be used for simultaneous clean-up and preconcentration of strongly and weakly acidic organic pollutants in real environmental water, which could not be achieved by single use of quaternary ammonium strong anion-exchange phase or weaker primary and secondary amine anion-exchange.

## 1. Introduction

Despite those great progresses achieved in improving precision and accuracy of analytical instruments, determination of analytes at trace level in complex matrices is still a huge challenge for direct instrumental analysis. At this time, the pretreatment step with the purpose of separation and/or enrichment of target analytes becomes a key prerequisite for accurate analysis. Even for high-sensitivity analyses, such as those employing liquid chromatograph-mass spectrometer/mass spectrometer (LC-MS/MS), proper sample preparation can be critical for minimizing matrix effects and concentrating analytes of interest [1,2]. Among many pre-treatment methods, solid phase extraction has been widely used due to its high incidence rate, simple operation, low solvent consumption, and good reproducibility [3,4,5,6,7].

The principle of solid phase extraction is that the target analyte can be retained on the adsorbent by the interaction between them with the aim of enrichment and separation. According to the mode of interaction, the adsorbent can be roughly classified into two types, a single-mode adsorbent, and a mixed adsorbent [8,9]. Single-mode adsorbents mainly include reversed phase, normal phase, and ion exchange adsorbents. The reversed phase and normal phase ones are often applied for retaining target analytes of non-polar, moderately polar to polar in the sample by hydrophobic interaction and polar interaction, respectively. Ion exchange adsorbent is mainly based on ion exchange interaction between the adsorbent and target analytes [10,11,12,13,14]. The disadvantage of single-mode adsorbents lies in that the mode of interaction is all the same for target analytes and other interferences. Thus, when the sample is very complex or contains many interference components, the performances in both removing interference components and obtaining good recoveries of target analytes are not optimistic. To solve the shortcomings of single-mode SPE, mixed-mode sorbents are designed to exhibit two or more primary interactions for the analyte retention, enabling greater cleanup selectivity and capacity of the extraction process. The combination of hydrophobic and ion exchange interactions is by far the most popular mixed mode, mainly including anion-exchange and cation-exchange mixed-mode sorbents, which have been used in highly efficient and selective extraction of acidic and basic compounds, respectively [15,16].

Nonsteroidal anti-inflammatory drugs (NSAIDs) are one of the most frequently used pain killer medicines, and NSAIDs cause toxicity in the environment even at very low concentration of ng L^−1^ to μg L^−1^. The presence of NSAIDs residues in natural water systems has raised major concerns for their potential risk to public health. Most NSAIDs contain a carboxylic acid group that can exist in protonated (neutral) state or in an ionized (charged) form. Therefore, mixed-mode SAX (SAX) sorbent such as Oasis MAX (pKa > 18) perform well in retaining and releasing weakly acidic NSAIDs at their deprotonation and protonation states, respectively. However, niflumic acid is a relatively strong acid (pKa = 1.68) that maintains ionization over the whole pH range investigated. As a result, mixed-mode SAX can be challenging as it is difficult to disrupt the ion-exchange interaction by only pH adjustment. Mixed-mode weak anion-exchange (WAX) sorbent with primary, secondary, and tertiary amine as functional groups introduces additional selectivity, which allows users to control the ionization of stationary phase by modifying the pH. In conclusion, mixed-mode SAX is for weakly acidic analytes while mixed-mode WAX is for strongly acidic analytes, since irreversible binding happens between a strong analyte and strong sorbent and it is difficult for weak sorbent to attract a weak analyte.

Compared to silica-based sorbents, polymer-based sorbents have wide pH range and no silanol interactions exists [17]. The Oasis mixed-mode WAX sorbent is a derivative of Oasis HLB copolymer, poly(N-vinylpyrrolidone-co-divinylbenzene), with piperazine ligands. SOLA WAX (Thermo Scientific, Waltham, MA, USA) polymeric SPE utilizes a hydrophobic backbone functionalized with a primary amine with a pKa of approximately 6. Fontanals et al. developed mixed-mode WAX sorbents based on poly(vinylbenzylchloride-co-divinylbenzene) microspheres modified with 1,2-ethylenediamine and piperazine moieties [18]. These WAX sorbents all need an additional modification step for introducing the WAX functionalities. Direct copolymerization of functional monomers undoubtedly provides the simplest approach for preparation of functionalized sorbents. Using this technique, a synthesized copolymer based on poly(N-vinylimidazole-co-divinylbenzene) was evaluated as a mixed mode WAX sorbent for SPE, since it contained an imidazole group with a pKa of approximately 7 [19]. Moreover, most existing strategies for the synthesis of such mixed-mode polymeric microspheres are based on classical emulsions stabilized by surfactants. In contrast, Pickering emulsions utilize solid microparticles or nanoparticles that localize at the interface between liquids as stabilizers to enhance the droplet lifetime, which afford higher stability, lower toxicity, and stimuli-responsiveness. Therefore, one-step Pickering emulsion polymerization can be considered attractive due to its easy preparation, enhanced stability, and various possibilities for preparing new materials [20,21,22].

In current work, we used a facile one-step Pickering emulsion polymerization for preparing poly(DEAEMA-co-DVB) microspheres that could simultaneously and efficiently extract strongly (niflumic acid) and weakly (diflunisal, naproxen, ketoprofen, mefenamic acid, and diclofenac) acidic NSAIDs. The developed poly(DEAEMA-co-DVB) sorbent contains a tertiary amine with a pKa value of around 10.7, which allows for control of the ionization of stationary phase by modifying the pH. Tertiary amine functionality can easily exchange strongly acidic NSAIDs anion. Furthermore, tertiary amine is more basic than primary amine (pKa ≈ 6), imidazole and pyridine, and therefore provides better anion-exchange (acid-base) interactions with those weak acidic NSAIDs. Therefore, our poly(DEAEMA-co-DVB) WAX can simultaneously enrich, purify, and analyze strongly and weakly acidic NSAIDs.

## 2. Materials and Methods

### 2.1. Chemicals and Materials

The reagents involved for synthesizing the polymer were divinylbenzene (DVB) and 2,2-azobisiobutyronitrile (AIBN) supplied by J&K Scientific (Beijing, China), nano-SiO_2_ (12 nm particle diameter) supplied by Sigma-Aldrich (St. Louis, MO, USA), and 2-(diethylamino)ethyl methacrylate (DEAEMA, 98.5%) as well as n-dodecanol (99.0%) from Tokyo Chemical Industry Co. (Tokyo, Japan). The pharmaceutical analytes were naproxen (NAP, 98%) from J&K Scientific (Beijing, China), ketoprofen (KEP, 98%) from Sigma-Aldrich (St. Louis, MO, USA), and niflumic acid (NIF,98%), diflunisal (DIF, 98%), mefenamic acid (MEF, 98%), and diclofenac (DIC, 98%) obtained from Accela Chem Bio (Shanghai, China). To remove inhibitors from DVB and DEAEMA, they were treated by passing through short columns containing 2 g of alumina-B (Santa Ana, CA, USA). The water used in all experiments was deionized water from a Milli-Q purification system (Billerica, MA, USA). The empty polypropylene SPE tubes (3 mL) with bio-fiber frits were supplied by Shenzhen Biocomma Technologies Co. (Shenzhen, China).

### 2.2. Preparation of Mixed-Mode WAX Microspheres

The mixed-mode WAX microspheres were synthesized via one-step Pickering emulsion polymerization procedure. First, 0.422 g of n-dodecanol, 22.5 mg of AIBN, 1.2 mL of toluene, 0.4 mL of DEAEMA, and 1.2 mL of DVB were mixed, followed by the treatment of nitrogen purge. Then, 120 mg of silica and 10 mL of water were mixed and vortexed for 1 min. Next, the mixed precursor solution was added to this solution, uniformly dispersed with a high-speed disperser, and placed in a sealed tube. The polymerization was carried out at 60 °C for 24 h. After polymerization, the polymer was washed with ethanol and dried. The solid was soaked in hydrofluoric acid for 24 h to etch away the silica, and then extracted with Soxhlet extraction for 12 h. The withdrawn material was drained, and a new WAX sorbent was obtained in this way.

### 2.3. Sample Collection

Water samples were collected from Xishan Reservoir (Dalian, China), part of the city water supply system, stored on ice and immediately transported to the laboratory. Then, the samples were filtered through 0.45 μm fibers to remove particulate matter, stored in clean glass bottles and kept in a refrigerator at 4 °C until SPE process.

### 2.4. Characterization of the Sorbent

The obtained materials were characterized by the following analytical techniques. Scanning electron microscopy (SEM) was performed on a JSM-7800F scanning electron microscope (JEOL, Tokyo, Japan). Nitrogen adsorption–desorption isotherms were performed at 77 K using a quantachrome Autosorb-iQ2 instrument (Quantachrome, Boynton beach, FL, USA). An approximately 0.1-g sample was degassed under vacuum at 80 °C for 12 h prior to measurement. The FT-IR spectra were 50 mg of poly(DEAEMA-co-DVB) was packed into 3 mL SPE tube. The tube was first conditioned with 3 mL of MeOH and 3 mL of H_2_O. After conditioning, the water sample was poured into the cartridge. The cartridge was first washed with 2 mL of MeOH/H_2_O (1:1), and then the target analytes were eluted out using 2 mL of NH_3_·H_2_O/MeOH (1:49). The extract was evaporated to dryness under nitrogen, reconstituted in 1 mL of the initial mobile phase and then filtered for HPLC-UV analysis.

### 2.5. Chromatographic Analysis

The chromatographic system was an Agilent 1200 series HPLC system (Santa Clara, CA, USA) equipped with a quaternary pump, an on-line degasser, an autosampler and a diode array detector (DAD). Chromatographic separations were performed on an Agilent ZORBAX Eclipse XDB-C18 column (5 μm, 150 mm × 4.6 mm i.d., Palo Alto, CA, USA). The mobile phase was composed of Solvent A (methanol) and Solvent B (20 mM ammonium acetate). The chromatographic separation was run as follows: a gradient from 30% to 75% of Solvent A in 20 min, then returned to initial condition in 0.5 min, and held for 3.5 min. All sample injection volumes were 20 μL. The column temperature was kept at 25 °C. The detection wavelengths were set at 230 nm for NIF, and 288 nm for NAP, KEP, DIF, MEF, DIC.

## 3. Results and Discussion

### 3.1. Preparation of the Poly(DEAEMA-co-DVB) Microspheres

**Figure 1:**
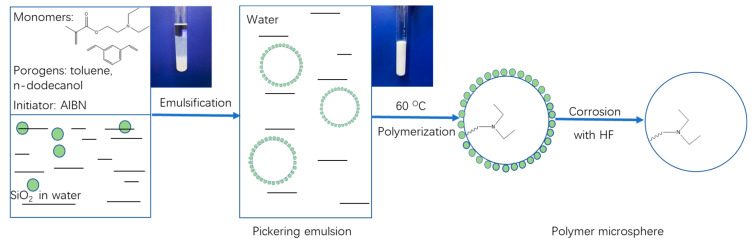
Schematic illustration for preparation of poly(DEAEMA-co-DVB) microspheres.

As shown in Figure 1, the poly(DEAEMA-co-DVB) microspheres were prepared using a SiO_2_-stabilized Pickering emulsion polymerization method. By self-organization of SiO_2_ at the interface of oil (monomers and porogens) and water, the stabilized oil-in-water Pickering emulsion droplets were formed and used as polymerization vessels to obtain the polymer/SiO_2_ composites by thermal radical polymerization. The SiO_2_-covered spherical particles were then etched using hydrofluoric acid to remove SiO_2_, and the poly(DEAEMA-co-DVB) microspheres were finally obtained. Here, the monomers DEAEMA and DVB afforded the weak anion-exchange and hydrophobic functionalities, respectively, and a combination of them successfully constructed the mixed-mode weak anion-exchange medium.

#### Characterization of the Poly(DEAEMA-co-DVB) Microspheres

**Figure 2:**
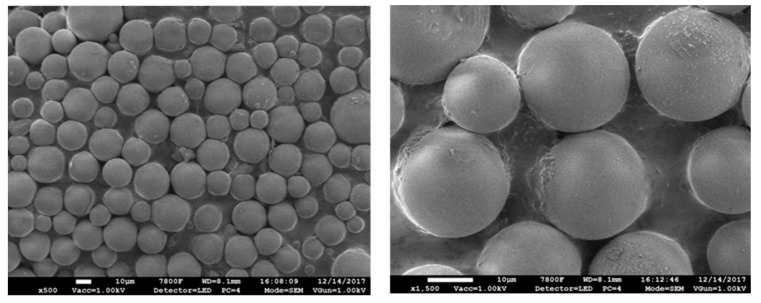
SEM images of poly(DEAEMA-co-DVB) microspheres.

A dose of 120 mg for silica nanoparticles was adopted with the aim of optimized uniformity and particle size. The synthesized solid material was subsequently characterized by SEM, nitrogen physical absorption, and FT-IR measurements. The SEM results shown in Figure 2 verify that most of the particles have spherical shape with the particle size range of 15–30 μm.

**Figure 3:**
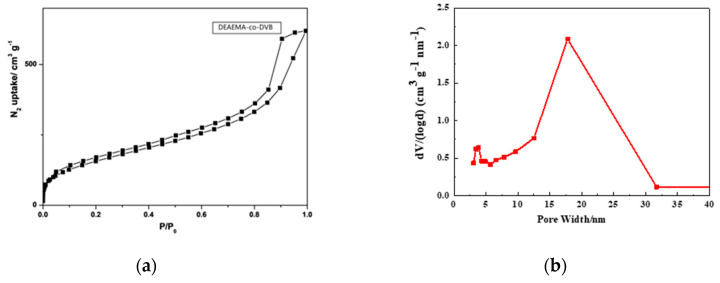
(**a**) N2 adsorption–desorption isotherms; and (**b**) pore size distribution of poly(DEAEMA-co-DVB) microspheres.

The N_2_ sorption–desorption isotherm gives the porous structure of poly(DEAEMA-co-DVB), as shown in Figure 3. The obvious hysteresis loop between absorption and desorption for capillary condensation demonstrates a mesoporous structure of the poly(DEAEMA-co-DVB). The pore size distribution analyzed by BJH is presented in Figure 3, which further proves that the mesopore size ranges from 10 to 30 nm.

**Figure 4:**
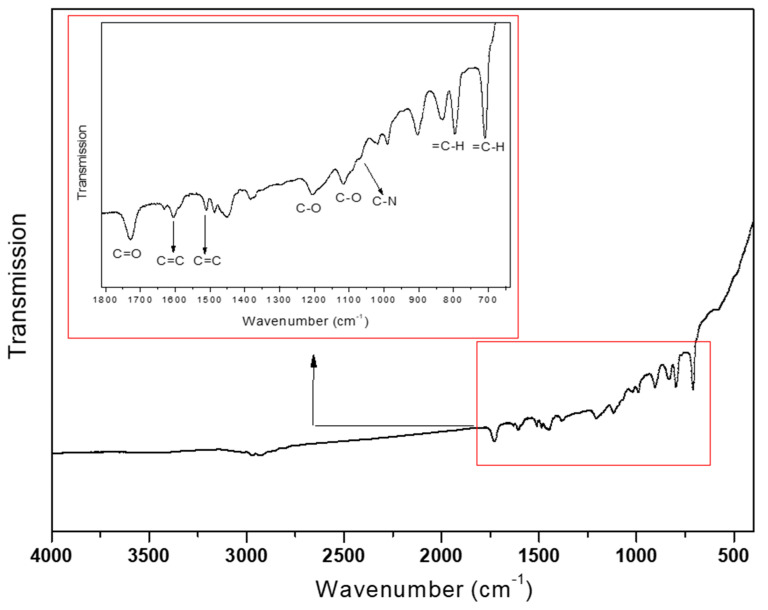
FTIR spectra of poly(DEAEMA-co-DVB) microspheres.

As presented in Table 1, the BET surface area and pore volume were calculated as 575 m^2^/g and 0.94 cm^3^/g, respectively, which is very beneficial for the adsorption of pharmaceutical molecules in solution. Additionally, the FT-IR was also carried out for identify the functional groups, and the results are given in Figure 4. The peak at 1066 cm^−1^ is ascribed as the stretching vibration of tertiary amine in poly(DEAEMA-co-DVB). The DVB as the copolymerized monomer is approved by the peaks at 1604 and 1510 cm^−1^ attributed to C=C, and the peaks at 796 and 710 cm^−1^ are attributed to =C–H of benzene ring. Moreover, the signal at 1727 cm^−1^ is testified as the C=O stretching of ester, which is different from the fact that carbonyl signal of –COOC=C appears in DEAEMA monomer, strongly implying that the vinyl-copolymerization process of DEAEMA and DVB was successful due to the disappearance of vinyl group. In addition, the existence of 1205 and 1118 cm^−1^ for C–O–C stretching was also assigned to the ester group. Thus, the IR test confirms the correct synthesis of poly(DEAEMA-co-DVB) material.

### 3.2. Optimization of SPE Procedures

We chose a group of acidic pharmaceuticals ranging from strongly acid to weak ones as target analytes (NAP, KEP, DIF, MEF, DIC, and NIF), and their molecular structures, log P, and pKa values are listed in Table 2. To obtain an ideal result in the real sample experiment, the optimized SPE protocol is necessary for the theoretical conditions, which can be applied for the real sample. The parameters that need to be evaluated involve the pH of the sample solution, the volume of sample solution (breakthrough volume), and the volume of elution solvent. All of the experiments for the SPE optimization except for the optimization of breakthrough volume were performed by loading 0.5 μg of NAP and 5 μg of KEP, DIF, MEF, DIC, and NIF in 10 mL of ultrapure water. One set of three samples was tested in parallel. Except for the condition being evaluated, all the others remained the same.

#### 3.2.1. Effect of Sample pH

**Figure 5:**
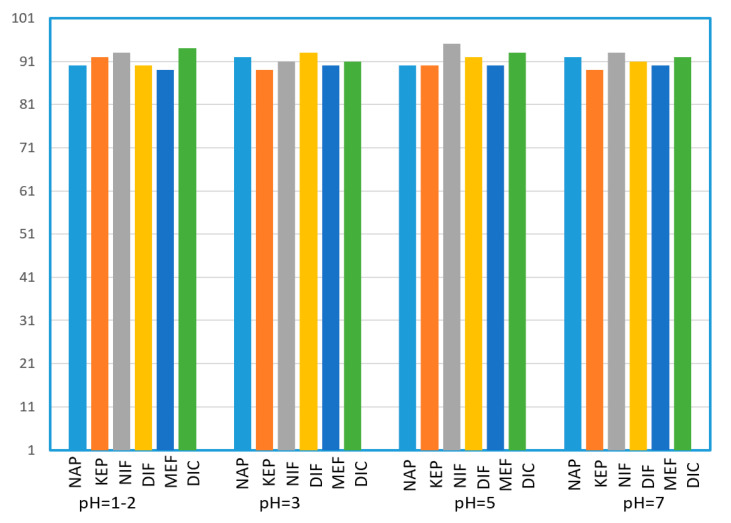
Effects of sample pH on the recoveries (%) of selected pharmaceuticals, *n* = 3.

Before conducting the experiment, we selected the sample pH of 1, 3, 5, and 7 for investigating its effect on recoveries of all pharmaceuticals. After loading the sample, the cartridges were washed with 4 mL of H_2_O/MeOH (1:1). In the pH range around pKa, small changes in pH provide major changes in ionization and retention. Higher pH above their pKa values leads to an increase in the degree of ionization of acidic targets, making the acidic drug mainly retained by ion-exchange mechanism. As the sample pH decreases, the degree of ionization of the acidic drug also decreases, but at this time the acidic target can be well retained on the column by hydrophobic interaction. In conclusion, in the entire pH range, the two complement interaction mechanisms can retain the acidic analytes well, in whatever form they exist. This was confirmed by the fact that the recoveries of all analytes varied little with the changes in the investigated pH range. As shown in Figure 5, most surface water samples are roughly neutral. Therefore, in the subsequent experiments, there was no need to adjust the sample pH before loading it on the SPE column.

#### 3.2.2. Effect of Elution Volume

**Figure 6:**
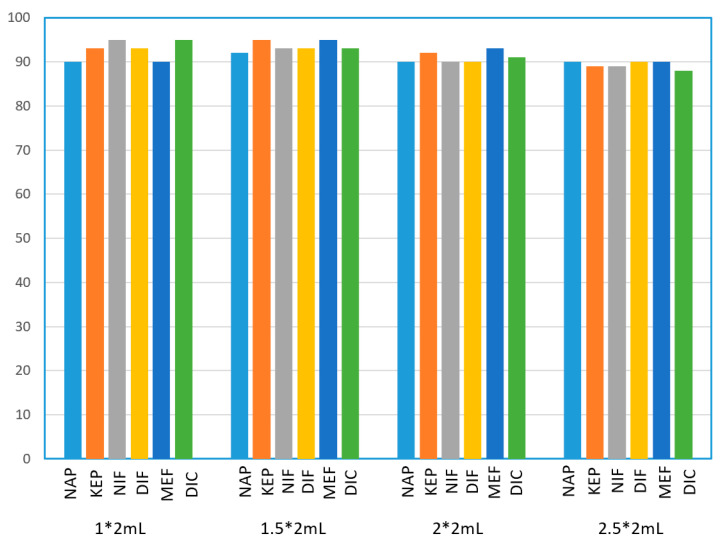
Effects of elution volume on the recoveries (%) of selected pharmaceuticals, *n* = 3.

To effectively elute an analyte that is being retained on a mixed-mode surface via two retention mechanisms, it is necessary to use an elution solvent that simultaneously disrupts both of the retention mechanisms. Therefore, methanol with 2% NH_3_·H_2_O was selected as the elution solvent to deprotonate the stationary phase. Theoretically, the amount of elution solvent is positively related to the elution ability; however, to minimize the volume of the elution solution and reduce the nitrogen purge time for subsequent experiment, the optimization experiments of elution volume, including 1 × 2, 1.5 × 2, 2 × 2, and 2.5 × 2 mL, were carried out. The experimental results are shown in Figure 6. As shown in Figure 6, we found that, even with the smallest 1 × 2 mL elution volume, the recoveries of all pharmaceuticals were still satisfying. Therefore, in the subsequent experiments, we chose 1 × 2 mL as the elution volume, which helped reduce the eluent volume and labor cost.

#### 3.2.3. Sample Breakthrough Volume

**Figure 7:**
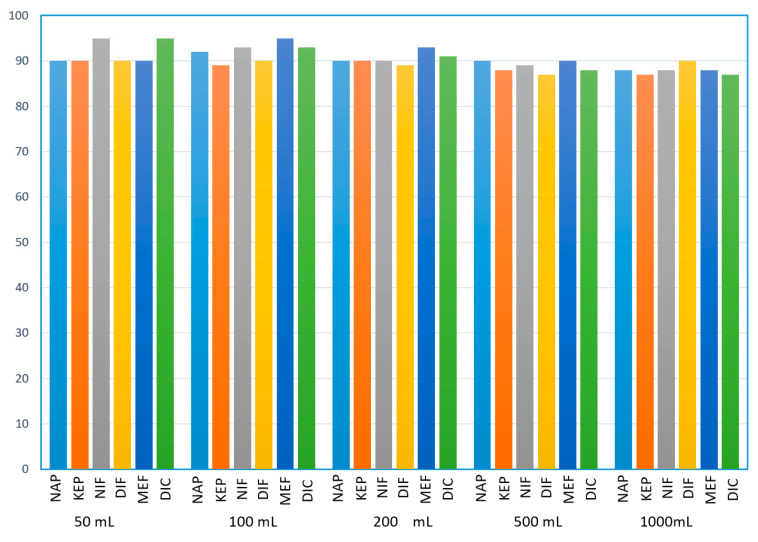
Effects of sample volume on the recoveries (%) of selected pharmaceuticals, *n* = 3.

Large volume water analysis is beneficial for a larger preconcentration factor and thus higher detection sensitivity. To determine the sample breakthrough volume, 50, 100, 200, 500, and 1000 mL volumes of ultrapure water containing 0.5 μg/L of NAP and 5 μg/L of KEP, DIF, MEF, DIC, and NIF were percolated through the column. The results in Figure 7 demonstrate excellent recoveries of all pharmaceuticals above 88% with a sample volume up to 1000 mL, implying that a theoretically 1000-fold preconcentration factor was achieved. However, due to an increased back pressure as a result of possible polymer swelling and particulate plugging, a 500 mL volume for real environmental samples was selected to avoid the complete block of SPE process.

### 3.3. Validation of the WAX Mixed-Mode SPE/HPLC-UV Method

The signal intensity (peak area) for each target analyte is proportional to its concentration within the range of 0.01–2.0 μg L^−1^ for NAP and 0.05–10.0 μg L^−1^ for KEP, DIF, MEF, DIC, and NIF with the correlation coefficients (r^2^) > 0.994. Using signal to noise method, the estimated LOD and LOQ were in the range of 0.002–0.025 and 0.003–0.088 μg L^−1^, respectively, as shown in Table 3. Additionally, the optimized SPE/HPLC-UV method displayed excellent recoveries of 88.3–101.8% for all pharmaceuticals in spiked samples. The intra-day and inter-day precision were also studied in terms of the relative standard deviation (RSD, *n* = 3) of the recoveries obtained by analyzing 500 mL of spiked samples within one day and during a period of three consecutive days, respectively. Moreover, the outstanding repeatability of good intra- and inter-day shown in Table 3 illustrate the feasibility of the proposed WAX mixed-mode SPE/HPLC-UV method for trace analysis of various acidic drugs, which offers an alternative for simultaneous detection of strongly and weakly acidic NSAIDs in real environmental water.

### 3.4. Analysis of Real Samples

We applied the established method for analysis of real environmental water samples. Our analysis results show that the six targets were not detectable in the reservoir water using the current established method. To test the applicability of our optimized method to real samples, spiked reservoir samples were used instead. As shown in Table 4, we added two spiked levels of the target analytes, one spiked at 0.05 μg/L of NAP and 0.5 μg/L of KEP, DIF, MEF, DIC, and NIF and another spiked at 0.5 μg/L of NAP and 5 μg/L of KEP, DIF, MEF, DIC, and NIF. The satisfactory recoveries of 88.3–101.7% show the reliability of our established method. We also compared the SPE performance of the laboratory-made WAX poly(DVB-co-DEAEMA) with a commercial C18 and MAX for extraction of six NSAIDs from spiked reservoir water. The 500-mL reservoir water sample was spiked at 0.05 μg/L of NAP and 0.5 μg/L of KEP, DIF, MEF, DIC, and NIF. Table 5 shows that, compared with C18, our current WAX gives much higher recoveries for all NSAIDs, which can be ascribed to the stronger retention from both a dual interaction mechanism and a high specific surface area.

For most pharmaceuticals, our results were comparable or even superior to Oasis MAX, especially for NIF. NIF is a strong acid that remains ionized across the whole pH range. Thus, NIF has a very strong interaction with the SAX Oasis MAX so that it is not possible to disrupt the ion exchange mechanism by pH adjustment, thus resulting in a low recovery of 30%. However, our WAX SPE utilized a hydrophobic backbone functionalized with a tertiary amine with a pKa of approximately 10.6, which allows easy control of the ionization of the stationary phase by modifying the pH. At the same time, a pKa of approximately 10.6 guaranteed that the stationary phase was positively charged across the investigated pH range. As a result, our WAX SPE can be applicable to both strongly and weakly acidic compounds. It was demonstrated that the laboratory-made poly(DVB-co-DEAEMA) can be applied to the extraction of NSAIDs from NIF (strongly acidic) to KEP, DIF, MEF, DIC, and NIF (weakly acidic), and the detection limit can be as low as 0.01 μg/L. In addition, our results are comparable to the performance of those mixed-mode SPE sorbents used in other previous reports. In conclusion, the laboratory-made poly(DVB-co-DEAEMA) can be an excellent mixed-mode SPE material for sample pretreatment of acidic organic pollutants in environmental samples.

## 4. Conclusions

For the first time, a mixed-mode WAX poly(DVB-co-DEAEMA) utilizing a hydrophobic backbone functionalized with a tertiary amine (with a pKa of approximately 10.6) was successfully used for SPE extraction of widely acidic pharmaceuticals (pKa values ranging from 1.68 to 4.45) from real water samples. This result was difficult to achieve by mixed-mode SAX and mixed-mode WAX containing weaker primary and secondary amines. The former cannot perform well for strong acids while the latter was not a good choice for weakly acidic analytes. In contrast to both, our mixed-mode WAX SPE can be successfully applied in simultaneous efficient enrichment of both strongly and weakly acidic NSAIDs with satisfying recoveries for the first time. The other importance of this work lies in that the functionalized sorbent was synthesized in a well-defined micro-sphere format by simple one-step Pickering emulsion polymerization. The method is process-simplified, as only one step was included in the fabrication. Finally, the application of our mixed-mode WAX not only allows selective extraction of the target analytes but also cleans up the sample, which are important particularly when the sample is complex and impurities can interfere with quantification.

## Figures and Tables

**Table 1:** Textural properties of the sorbent.

	S_BET_ (m^2^ g^−1^)	Pore Volume (cm^3^ g^−1^)	Pore Size (nm)
Poly(DVB-co-DEAEMA)	575	0.94	10–30

**Table 2:** The physical properties and structures of selected NSAIDs.

Pharmaceutical	CAS Number	Structure	pKa	Log P	Water Solubility (mg L^−1^)
niflumic acid	4394-00-7	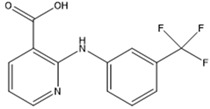	1.68	4.43	19
naproxen	22204-53-1	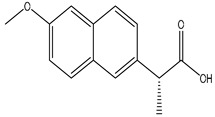	4.15	3.18	15.9
diflunisal	22494-42-4	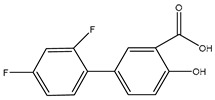	3.3	4.44	14.5
diclofenac	15307-86-5	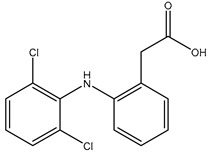	4.15	4.51	2.37
ketoprofen	22071-15-4	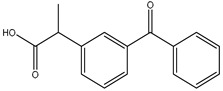	4.45	3.12	51
mefenamic acid	61-68-7	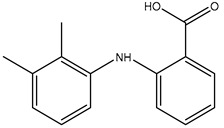	4.2	5.12	20

**Table 3:** Liner regression, LODs, LOQs, and RSDs data for the poly(DEAEMA-co-DVB) based WAX SPE/HPLC-UV method.

Analyte	Liner Range (μg L^−1^)	Correlation of Determination (R^2^)	LOD ^a^ (μg L^−1^)	LOQ ^a^ (μg L^−1^)	Intra-Day RSD (%, n = 3)	Inter-Day RSD (%, n = 3)
naproxen	0.01–10.0	0.995	0.011	0.032	0.3	3.3
ketoprofen	0.05–10.0	0.994	0.025	0.044	0.5	2.7
diflunisal	0.05–10.0	0.996	0.018	0.076	0.9	3.1
mefenamic acid	0.05–10.0	0.995	0.016	0.088	0.9	2.8
diclofenac	0.05–10.0	0.997	0.008	0.006	1.3	4.0
niflumic acid	0.05–10.0	0.997	0.002	0.003	2.1	3.3

^a^ LODs and LOQs were estimated as the concentration where S/N = 3 and 10, respectively.

**Table 4:** Recoveries (%) and RSDs after the WAX based SPE of 500 mL of reservoir water samples at two different spiked levels.

Analyte	Spiked Levels ^a^			
	(1)		(2)	
	Eluate ^b^	RSD%	Eluate	RSD%
naproxen	101.3	2.7	92.0	3.6
ketoprofen	93.3	1.8	101.7	4.4
diflunisal	89.5	7.7	98.4	5.6
mefenamic acid	92.4	1.4	93.6	7.2
diclofenac	96.6	2.2	88.3	2.7
niflumic acid	90.6	4.6	90.1	3.5

^a^ (1) Spiked at 0.05 μg/L of NAP and 0.5 μg/L of KEP, DIF, MEF, DIC, and NIF; and (2) Spiked at 0.5 μg/L of NAP and 5 μg/L of KEP, DIF, MEF, DIC, and NIF. ^b^ Eluate was obtained using 2 mL of 2% NH_3_·H_2_O/MeOH as eluent.

**Table 5:** Comparison of recovery performance between poly(DEAEMA-co-DVB) with commercial SPE columns.

Analyte	Poly(DEAEMA-co-DVB) ^a^	Oasis MAX ^a^	C18 ^a^
naproxen	90	93	70
ketoprofen	98	93	73
diflunisal	92	90	66
mefenamic acid	89	88	83
diclofenac	88	90	62
niflumic acid	93	7	69

^a^ Spiked at 0.05 μg/L of NAP and 0.5 μg/L KEP, DIF, MEF, DIC, and NIF.
